# Real-world obesity prevalence and history in 79 271 patients receiving systemic anticancer therapy across 13 cancer types in England (2013-2023)

**DOI:** 10.1016/j.esmorw.2026.100700

**Published:** 2026-04-24

**Authors:** V. Perletta, S. Kulkarni, Z. Wang, J.W. Tomlinson, J. Hippisley-Cox, G.S. Collins, A.K. Clift, D. Dodwell, S.R. Lord

**Affiliations:** 1 Department of Oncology, University of Oxford, Oxford, UK; 2 Nuffield Department of Population Health, University of Oxford, Oxford, UK; 3 Oxford Centre for Diabetes, Endocrinology & Metabolism and NIHR Oxford Biomedical Research Centre, University of Oxford, Oxford, UK; 4 Wolfson Institute of Population Health, Queen Mary University of London, London, UK; 5 Department of Applied Health Sciences, School of Health Sciences, College of Medicine and Health, University of Birmingham, Birmingham, UK; 6 NIHR Birmingham Biomedical Research Centre, Birmingham, University Hospitals Birmingham NHS Foundation Trust and University of Birmingham, Birmingham, UK; 7 Department of Surgery & Cancer, Imperial College London, London, UK

**Keywords:** body mass index, obesity, prognosis, real-world evidence, systemic anticancer therapy

## Abstract

**Background:**

Obesity is recognized as a risk factor for several cancer types and is linked with different patient outcomes. However, the extent to which obesity prevalence at treatment initiation differs from lifetime (ever) obesity exposure remains unclear.

**Materials and methods:**

In this descriptive real-world study, we analysed longitudinal body mass index (BMI) records from the QResearch general practice database linked to the National Health Service England Systemic Anti-Cancer Therapy dataset including patients with a first systemic treatment in 2013-2023. We calculated age-standardised obesity prevalence at first treatment and lifetime obesity prevalence based on BMI ≥30 kg/m^2^ at treatment initiation and from historic measurements for patients across 13 cancer types.

**Results:**

In total, 79 271 patients were included (median age 66.5 years at first treatment, 54.2% female, and 89.0% white ethnicity). Age-standardised obesity prevalence at first treatment was 26.4% [95% confidence interval (CI) 26.0% to 26.9%] for all cancers, ranging from 13.7% (95% CI 11.6% to 15.9%) for pancreatic cancer to 36.3% (95% CI 29.9% to 42.7%) for uterine cancer. Lifetime obesity prevalence was 53.5% (95% CI 53.2% to 53.9%) for all cancers, ranging from 51.1% (95% CI 50.3% to 52.0%) for lung cancer to 63.0% (95% CI 58.8% to 67.2%) for hepatocellular carcinoma.

**Conclusions:**

We found that approximately one in four cancer patients in England were living with obesity at the start of systemic treatment, while half had a history of obesity. Reliance on BMI at treatment initiation for cancer prognostication substantially underestimates lifetime exposure to obesity with implications for precision medicine and outcomes research.

## Highlights

• This study assessed real-world obesity prevalence in 79 271 patients receiving systemic anticancer therapy in England in 2013-2023.
• Obesity prevalence at treatment start substantially underestimated lifetime obesity exposure.
• Lifetime obesity was identified in more than half of patients receiving systemic therapy across all 13 cancer types.
• Obesity prevalence at treatment start varied by cancer type, age, sex, ethnicity, and deprivation.
• Findings highlight the need to incorporate lifetime exposure to obesity into prognostic cancer research.

## Introduction

Obesity prevalence has been increasing rapidly over the last 30 years,^1^ contributing to the growing worldwide incidence of cancer.^2^ Forecasts indicate that over 2 million new cancer cases globally will be attributable to obesity by 2070, up from half a million in 2012.^3^ Consequently, clinicians will increasingly encounter patients with obesity-related cancers (including breast, bowel, pancreatic, liver, oesophageal, and uterine cancers^4^) and patients with other cancers presenting concomitantly with obesity or obesity-associated comorbidities such as insulin resistance and metabolic dysfunction.^5^

Obesity in people diagnosed with cancer has been associated with adverse clinical outcomes, including higher rates of surgical complications,^6^ adverse drug effects,^7^ and disease recurrence.^8^ Some cancer patients with obesity are reported to receive lower doses of chemotherapy^9^ despite clinical guideline recommendations discouraging reductions based on body weight or body surface area alone.^10^ As use of fixed-dose monoclonal antibodies and targeted small molecules becomes standard practice,^11^ these patients may again face an increased risk of suboptimal dosing. Conversely, emerging evidence suggests that cancer patients with obesity might respond better to immunotherapy and other systemic cancer treatments.^12^^,^^13^

Personalized treatment approaches may be required for cancer patients with obesity to ensure safety and effectiveness^14^; however, the scale of this need is unclear due to limited obesity prevalence data in real-world populations. Two existing reviews of obesity prevalence in patients receiving systemic anticancer therapy involve only clinical trials,^15^^,^^16^ in which obesity is likely underrepresented. As Vaidya et al.^16^ also noted, the included studies considered obesity only at treatment initiation, and the absence of historical obesity was a key limitation. Due to cancer-related weight loss and ageing,^17^ lifetime exposure (i.e. ever exposed) to obesity was almost certainly underestimated. Whereas obesity measured at treatment initiation likely reflects disease stage or metabolic consequences of cancer, lifetime obesity better captures pre-diagnostic exposure and disease risk.^18^

A contemporary real-world estimate of obesity prevalence and history in patients receiving systemic anticancer therapy is needed to clarify burden and inform health care provision. We therefore analysed large-scale linked electronic health record (EHR) data containing longitudinal body mass index (BMI) measurements for patients with 1 of 13 cancer types receiving first systemic therapy in England in 2013-2023. We report observed, age-standardised, and lifetime obesity prevalence by cancer type, age, sex, ethnicity, deprivation, and region.

## Materials and methods

### Study type, data sources, and definitions

This descriptive real-world study used individual-level EHR data from the QResearch primary care database (including >1500 general practices in England), linked to Hospital Episode Statistics (HES; Admitted Patient Care and Outpatients), and the National Cancer Registrations, Systemic Anti-Cancer Therapy (SACT), and Civil Registration of Deaths datasets. These datasets have all been extensively used and validated for epidemiological research.^19^, ^20^, ^21^, ^22^, ^23^, ^24^ The study period was 1 January 2013 to 31 May 2023, aligning with SACT dataset availability.

We included adults registered at QResearch practices for at least 1 year with a first diagnosis of 1 of 13 common cancer types (breast, prostate, lung, bowel, malignant melanoma, kidney, pancreatic, bladder, gastroesophageal, ovarian, hepatocellular, non-Hodgkin lymphoma, or uterine cancer) recorded in either QResearch, HES, or the Cancer Registry; a first systemic treatment recorded in SACT; and valid BMI measurements (validation rules below). Due to Cancer Registry censoring, diagnoses from 2021 were identified in QResearch and HES only, with 98% of eligible patients estimated to have been included (Supplementary Figure S1, available at https://doi.org/10.1016/j.esmorw.2026.100700).

**Figure 1:**
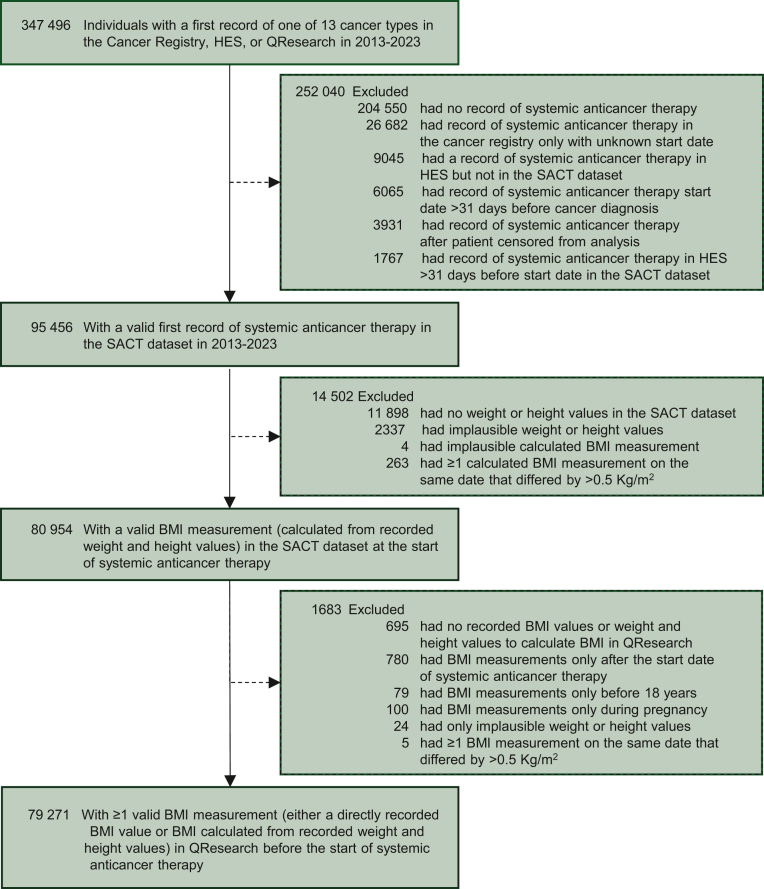
**Flow diagram showing number of individuals with cancer undergoing systemic anticancer therapy and with valid BMI measurements who were included in the study sample.** BMI, body mass index; HES, hospital episode statistics, SACT, systemic anticancer therapy.

Index date for each individual was the first systemic therapy regimen start date recorded in SACT, including records from up to 31 days before first cancer date,^25^ until study period end, death, or general practice deregistration. Because SACT records before 2017 are incomplete,^26^ some first treatment records in SACT may correspond to subsequent administrations. We therefore cross-checked systemic therapy dates captured in HES and excluded patients with evidence of an administration >31 days before the start date recorded in SACT (<2% patients excluded; Figure 1).

Study variable definitions and code lists are available at https://www.qresearch.org/data/qcode-group-library/. Cancer diagnoses were identified using International Classification of Diseases, Tenth Revision codes in the Cancer Registry and HES and Systematized Nomenclature of Medicine Clinical Terms in QResearch. Data regarding age, sex, Townsend deprivation fifths (1 least to 5 most deprived), and region of England were obtained from QResearch. Ethnicity was ascertained from QResearch, and missing data were supplemented by Cancer Registry, HES, or SACT records.^27^ Longitudinal BMI measures were either directly recorded or calculated from weight and height in QResearch and SACT and validated using established rules excluding implausible or inconsistent values and measurements during childhood or pregnancy (Supplementary Table S1, available at https://doi.org/10.1016/j.esmorw.2026.100700).^28^^,^^29^

### Statistical analysis

Observed obesity prevalence at first treatment was estimated as the percentage of patients with BMI ≥30 kg/m^2^ (World Health Organization definition^30^) among those with a valid BMI recorded in SACT on the index date. Age-standardised obesity prevalence was calculated to enable comparisons across cancer types accounting for potential confounding by age. We applied direct standardisation (age groups 18-54, 55-64, 65-74, and ≥75 years) referencing the 2022 mid-year population statistics for England,^31^ as used in the latest Health Survey for England report of obesity in England where prevalence is estimated at 29% of the adult population.^32^ History of obesity (lifetime prevalence) was determined using each patient’s maximum recorded BMI from all valid QResearch measurements before first treatment and the BMI recorded at first treatment in SACT, thereby capturing whether a patient has ever been exposed to obesity.

Prevalence and history of obesity estimates are reported as a percentage with 95% confidence interval (CI) for the overall sample of patients receiving systemic anticancer therapy with complete BMI data, separately for each cancer type and within prespecified subgroups of age, sex, ethnicity, and deprivation, as well as region. No missing values were imputed. Frequencies of <10 patients and corresponding percentages are not reported. Analyses were executed in Stata/MP 18.0 per the study protocol.

### Sensitivity analyses

Two sensitivity analyses assessed the stability of results. First, we implemented recently defined ethnicity-specific cutoffs for BMI, lowering the threshold for obesity in black, Asian, and other minority ethnicity groups to ≥27.5 kg/m^2^.^33^ Second, we excluded individuals with diagnoses of second cancers within 5 years, due to possible heterogeneity in patient and treatment characteristics.

### Ethics approval and consent to participate

The project was independently peer-reviewed and approved by the QResearch Scientific Board (reference: OX183). Ethical approval for the QResearch database is obtained annually from East Midlands—Derby Research Ethics Committee (reference: 18/EM/0400). General practitioners consent to take part in QResearch, and patients who do not wish their data to be included can opt out via the National Data Opt-Out (NDOO) service. This study was carried out in accordance with the Declaration of Helsinki.

### Reporting guidelines

We prepared the manuscript following the ESMO-GROW checklist.^34^

## Results

### Study sample derivation and characteristics

We identified 95 456 patients with a first cancer record and valid first systemic therapy record in the SACT dataset between 2013 and 2023. Of these, 79 271 (83.0%) had a valid BMI measurement recorded at first treatment and ≥1 valid BMI measurement taken up to first treatment recorded in QResearch (Figure 1). There were slight differences in the characteristics of patients with and without valid BMI measurements, with completeness on average being lower in individuals ≥75 years (77.6% complete), men (80.3% complete), some regions of England (lowest in the North East at 72.6% complete), and some cancer types (lowest in prostate cancer at 58.7% complete) (Supplementary Table S2, available at https://doi.org/10.1016/j.esmorw.2026.100700).

Study sample characteristics at first treatment are tabulated for overall sample (Table 1) and by cancer type (Supplementary Table S3, available at https://doi.org/10.1016/j.esmorw.2026.100700). Patients with breast cancer were most frequent in the sample (*n* = 18 859; 23.8%), followed by those with bowel cancer (*n* = 14 831; 18.7%), lung cancer (*n* = 13 298; 16.8%), and non-Hodgkin lymphoma (*n* = 7425; 9.4%). Patients with uterine (*n* = 472; 0.6%), hepatocellular (*n* = 511; 0.6%), and kidney (*n* = 1505; 1.9%) cancer were least frequent in the sample. These relative prevalences appropriately reflect the epidemiology of cancers commonly treated with systemic therapy in England.^35^

**Table 1:** Characteristics of the study sample at the start of systemic therapy

Patient characteristic at systemic therapy start	*N* = 79 271
Age at treatment start, median (25th, 75th)	66.5 (56.9, 74.0)
Age group (years) at treatment start, *n* (%)	
18-54	16 704 (21.1%)
55-64	19 420 (24.5%)
65-74	25 823 (32.6%)
75+	17 324 (21.9%)
Sex, *n* (%)	
Female	42 936 (54.2%)
Male	36 335 (45.8%)
Ethnicity, *n* (%)	
White	70 584 (89.0%)
Indian	1127 (1.4%)
Pakistani	698 (0.9%)
Bangladeshi	423 (0.5%)
Other Asian	820 (1.0%)
Caribbean	1034 (1.3%)
Black African	1077 (1.4%)
Chinese	303 (0.4%)
Other	2036 (2.6%)
Missing	1169 (1.5%)
Townsend deprivation fifth, *n* (%)	
1 (least deprived)	25 197 (31.8%)
2	19 470 (24.6%)
3	14 345 (18.1%)
4	11 015 (13.9%)
5 (most deprived)	8846 (11.2%)
Missing	398 (0.5%)
Region, *n* (%)	
East Midlands	1526 (1.9%)
East of England	3318 (4.2%)
London	15 356 (19.4%)
North East	2235 (2.8%)
North West	17 935 (22.6%)
South Central	11 102 (14.0%)
South East	8993 (11.3%)
South West	7707 (9.7%)
West Midlands	8951 (11.3%)
Yorkshire & Humber	2148 (2.7%)
Cancer type, *n* (%)	
Breast	18 859 (23.8%)
Bowel	14 831 (18.7%)
Lung	13 298 (16.8%)
Non-Hodgkin lymphoma	7425 (9.4%)
Gastroesophageal	6349 (8.0%)
Prostate	5441 (6.9%)
Pancreas	3185 (4.0%)
Ovarian	3140 (4.0%)
Bladder	2532 (3.2%)
Malignant melanoma	1723 (2.2%)
Kidney	1505 (1.9%)
Hepatocellular	511 (0.6%)
Uterine	472 (0.6%)

Median age at regimen start was 66.5 years overall, ranging from 56.7 years (breast) to 72.2 years (prostate) across cancer types. While 42 936 (54.2%) patients overall were female, most patients with non–sex-specific cancers were male. Most patients were white (*n* = 70 584; 89.0%), ranging from 79.7% (uterine) to 96.2% (malignant melanoma) across cancer types. Only 8846 (11.2%) patients overall were from the most deprived areas of England (i.e. highest fifth of the Townsend score), ranging from 5.9% (malignant melanoma) to 17.0% (hepatocellular carcinoma) across cancer types. Patients receiving systemic therapy from all regions of England were included, with the North West region most highly represented overall (*n* = 17 935; 22.6%). This was consistent across most cancer types, except for hepatocellular and uterine cancers, where the highest percentage of patients was from the London region (32.3% and 28.0%, respectively).

### Observed, age-standardised, and lifetime prevalence of obesity by cancer type

The observed, age-standardised, and lifetime prevalence of obesity in the overall sample of cancer patients receiving a first systemic therapy was 25.2% (95% CI 24.9% to 25.5%), 26.4% (95% CI 26.0% to 26.9%), and 53.5% (95% CI 53.2% to 53.9%), respectively (Table 2).

**Table 2:** Observed and age-standardised at first treatment and lifetime prevalence (% [95% CI]) of obesity in systemic anticancer therapy patients

Prevalence at systemic therapy start (observed and age standardised) and lifetime history of obesity (BMI ≥30 kg/m^2^) [% (95% CI)]					
	*n* / *N*	Observed	Age-standardised	*n* / *N*	Lifetime
All patients	19 958/79 271	25.2% [24.9-25.5%]	26.4% [26.0-26.9%]	42 422/79 271	53.5% [53.2-53.9%]
Uterine	181/472	38.3% [34.0-42.7%]	36.3% [29.9-42.7%]	290/472	61.4% [57.0-65.8%]
Melanoma	542/1723	31.5% [29.3-33.6%]	33.7% [30.5-37.0%]	989/1723	57.4% [55.1-59.7%]
Breast	6484/18 859	34.4% [33.7-35.1%]	33.2% [32.5-33.9%]	10 089/18 859	53.5% [52.8-54.2%]
Kidney	415/1505	27.6% [25.3-29.8%]	29.0% [25.3-32.8%]	912/1505	60.6% [58.1-63.1%]
Prostate	1433/5441	26.3% [25.2-27.5%]	28.7% [23.9-33.5%]	2833/5441	52.1% [50.7-53.4%]
Ovarian	791/3140	25.2% [23.7-26.7%]	27.2% [25.0-29.4%]	1679/3140	53.5% [51.7-55.2%]
Bladder	639/2532	25.2% [23.5-26.9%]	26.9% [23.2-30.6%]	1355/2532	53.5% [51.6-55.5%]
Hepatocellular	147/511	28.8% [24.8-32.7%]	24.1% [17.9-30.3%]	322/511	63.0% [58.8-67.2%]
Non-Hodgkin lymphoma	1719/7425	23.2% [22.2-24.1%]	23.8% [22.3-25.2%]	4155/7425	56.0% [54.8-57.1%]
Bowel	3415/14 831	23.0% [22.3-23.7%]	23.5% [22.4-24.5%]	7760/14 831	52.3% [51.5-53.1%]
Lung	2564/13 298	19.3% [18.6-20.0%]	20.2% [18.8-21.6%]	6798/13 298	51.1% [50.3-52.0%]
Gastroesophageal	1220/6349	19.2% [18.2-20.2%]	19.1% [17.4-20.7%]	3462/6349	54.5% [53.3-55.8%]
Pancreas	408/3185	12.8% [11.6-14.0%]	13.7% [11.6-15.9%]	1778/3185	55.8% [54.1-57.5%]

Age standardisation by direct method (age groups 18-54, 55-64, 65-74, and ≥75 years) was carried out using the 2022 mid-year population statistics for England as the reference population.^31^

BMI, body mass index; CI, confidence interval; *n* / *N,* number of patients with obesity (BMI ≥30) / total patients with a BMI value.

By cancer type, the age-standardised prevalence of obesity at first treatment was highest in patients with uterine cancer (36.3%, 95% CI 29.9% to 42.7%), followed by malignant melanoma (33.7%, 95% CI 30.5% to 37.0%) and breast cancer (33.2%, 95% CI 32.5% to 33.9%). Age-standardised prevalence of obesity was lowest in pancreatic cancer (13.7%, 95% CI 11.6% to 15.9%), followed by gastroesophageal cancer (19.1%, 95% CI 17.4% to 20.7%), lung cancer (20.2%, 95% CI 18.8% to 21.6%), bowel cancer (23.5%, 95% CI 22.4% to 24.5%), and non-Hodgkin lymphoma (23.8%, 95% CI 22.3% to 25.2%).

**Figure 2:**
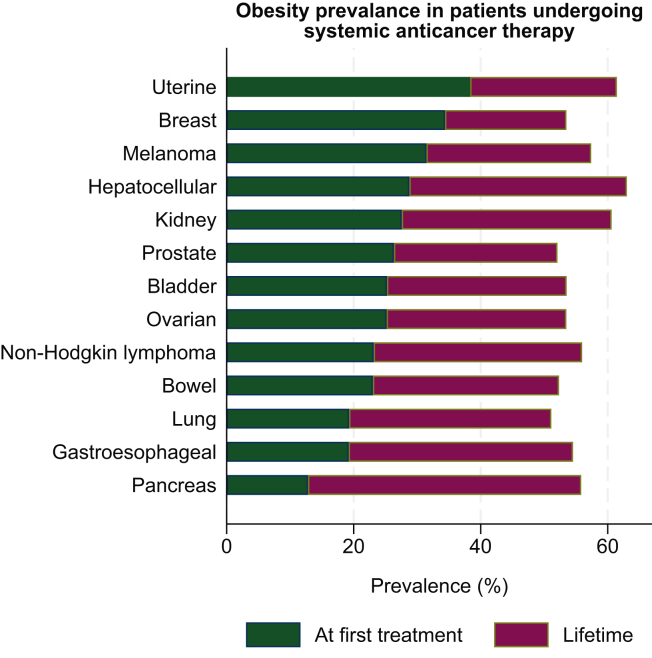
**Observed at first treatment and lifetime prevalence (%) of obesity in systemic anticancer therapy patients, by cancer type.** Plot shows observed obesity prevalence at first systemic therapy (green bar), and the percentage point increase in the obesity proportion when including patients with historic records of obesity (purple green bar).

Lifetime prevalence of obesity estimates for all cancer types exceeded over 50% of patients, estimated at 53.5% (95% CI 53.2% to 53.9%) for the overall sample and ranging from 51.1% (95% CI 50.3% to 52.0%) for lung cancer to 63.0% (95% CI 58.8% to 67.2%) for hepatocellular carcinoma. Compared with obesity prevalence at first treatment, lifetime prevalence of obesity was higher on average by 28.3 percentage points (95% CI 28.0 to 28.7), ranging from a 19.1 (95% CI 18.6 to 19.7) percentage point increase for breast cancer to a 43.0 (95% CI 41.3 to 44.7) percentage point increase for pancreatic cancer (Figure 2).

### Observed prevalence and history of obesity by age, sex, ethnicity, deprivation, and region

**Figure 3:**
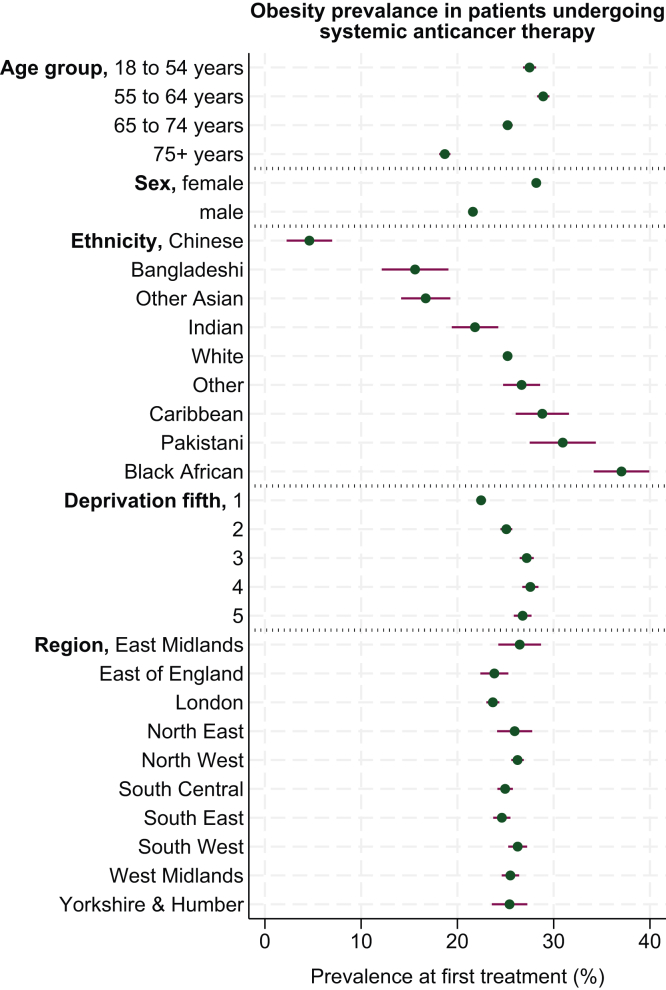
**Observed obesity prevalence (%) at first treatment for the overall sample of systemic anticancer therapy patients, by subgroups of age, sex, ethnicity, deprivation and region of England.** Plot shows obesity prevalence (green dot) with 95% confidence interval (purple error bar). Deprivation is based on the Townsend score, divided into fifths from 1 to 5, where 1 corresponds to the ‘least’ and 5 corresponds to the ‘most’ deprived areas of England.

Observed prevalence of obesity at first treatment estimates across subgroups of age, sex, ethnicity, deprivation, and region are shown in Figure 3 (overall) and Supplementary Figures S2-S6 (within each cancer type), available at https://doi.org/10.1016/j.esmorw.2026.100700. Age-standardised estimates were not computed due to insufficient event counts.

By age, patients aged ≥75 years had the lowest prevalence of obesity at first systemic therapy versus all other age groups [18.7% (95% CI 18.1% to 19.3%) versus 27.5% (95% CI 26.8% to 28.2%), 28.9% (95% CI 28.3% to 29.6%), and 25.2% (95% CI 24.7% to 25.7%) for ages 18-54, 55-64, and 65-74 years, respectively], which was generally observed across all cancer types (Supplementary Figure S2).

By sex, men had lower obesity prevalence (21.6%, 95% CI 21.2% to 22.0%) than women (28.2%, 95% CI 27.8% to 28.6%) overall, although this finding was not consistent for all cancer types, including breast cancer [40.3% (95% CI 31.5% to 49.2%) in men versus 34.3% (95% CI 33.7% to 35.0%) in women] and gastroesophageal cancer [19.9% (95% CI 18.7% to 21.0%) in men versus 17.5% (95% CI 15.7% to 19.3%) in women] (Supplementary Figure S3).

By ethnicity, Chinese patients were estimated to have the lowest prevalence of obesity (4.6%, 95% CI 2.3% to 7.0%), and black African patients were estimated to have the highest prevalence (37.0%, 95% CI 34.2% to 39.9%). Broadly similar patterns in ethnicity data were observed for patients with breast and bowel cancer; however, for most other cancer types, there were insufficient patient numbers to estimate ethnicity-specific values (Supplementary Figure S4).

By deprivation, obesity prevalence was higher in the most (26.8%, 95% CI 25.9% to 27.7%) versus the least deprived fifth (22.5%, 95% CI 21.9% to 23.0%) for the overall sample, and this was also demonstrated in breast cancer (39.1%, 95% CI 37.1% to 41.2% versus 30.3%, 95% CI 29.1% to 31.4%, respectively), ovarian cancer (31.8%, 95% CI 26.1% to 37.5% versus 20.6%, 95% CI 18.2% to 23.1%, respectively), and non-Hodgkin lymphoma (26.2%, 95% CI 22.9% to 29.4% versus 20.6%, 95% CI 19.0% to 22.1%, respectively) (Supplementary Figure S5).

By region, no clear or consistent regional differences were observed in obesity rates (Supplementary Figure S6).

History of obesity estimates across subgroups of age, sex, ethnicity, deprivation, and region are shown in Supplementary Figure S7, available at https://doi.org/10.1016/j.esmorw.2026.100700, with broadly similar findings.

### Sensitivity analyses

Implementing ethnicity-specific cutoffs for BMI had marginal impact on the prevalence of obesity observed at first treatment for the overall sample, which was estimated at 26.2% (95% CI 25.9% to 26.5%). However, there was on average a 15.3 (95% CI 14.4-16.3) percentage point difference in the obesity prevalence for patients from black, Asian, and other minority ethnicity groups (Supplementary Table S4, available at https://doi.org/10.1016/j.esmorw.2026.100700).

Results excluding patients with second cancers from the analysis differed little from those estimated in the main analysis (Supplementary Table S5, available at https://doi.org/10.1016/j.esmorw.2026.100700).

## Discussion

### Key findings and importance

In this large descriptive real-world study, approximately one in four patients with cancer were living with obesity at first systemic therapy. However, there were wide differences across cancer types and demography, and obesity prevalence was strikingly higher (increasing to one in two) when incorporating historic BMI information. To our knowledge, this is the first study to quantify the vast underestimation of lifetime obesity exposure when relying on BMI at first systemic therapy. Our findings suggest significant implications for understanding the relationship between obesity and cancer outcomes, especially for precision medicine. Failure to account for historic obesity introduces exposure misclassification, which may limit the accuracy of prognostic cancer models.

At first systemic treatment, we observed lower obesity prevalence for pancreatic, gastroesophageal, lung, and bowel cancers and non-Hodgkin lymphoma, which commonly present with cachexia or reduced dietary intake. We observed higher obesity prevalence for uterine, breast cancer, and malignant melanoma. Uterine and breast cancers are established obesity-related cancers, and ascites may increase BMI. The role of obesity, if any, in the development of malignant melanoma is unclear^4^; however, BMI has been shown to correlate with increased Breslow thickness (a prognostic indicator used in melanoma staging),^12^ thus, it could be that melanoma patients with higher BMI are more often candidates for systemic therapy.

Key results from obesity prevalence subgroup analyses by age, sex, ethnicity, deprivation, and region found that patients aged ≥75 years had consistently lower obesity at first systemic therapy and patients from the most deprived areas of England had higher levels of obesity. Studies on the life-course trajectory of BMI have reported that weight gain peaks between the ages of 50 to 69 and starts declining after the age of 70.^17^ Obesity and deprivation are strongly linked, with the latest Health Survey for England report (2022) estimating that the prevalence of obesity or being overweight is 12 percentage points higher in the most deprived areas in England than in areas regarded as least deprived.^32^

Our finding that half of patients undergoing systemic anticancer therapy may have a history of obesity is stark and demonstrates that assessing obesity only at treatment initiation substantially underestimates lifetime exposure. Whereas the link between obesity and cancer development is well established, how obesity relates to cancer outcomes remains uncertain. Some reports suggest that obesity may have a converse protective effect on cancer survival that may also be specific to certain systemic cancer treatments.^12^^,^^13^ However, although preclinical studies have suggested a potential biological basis for the ‘obesity paradox,’ it has also been argued that methodological nuances in obesity definitions and study population selection may also contribute.^18^ Our study promotes caution in the interpretation of research that has evaluated outcomes in patients with and without obesity at point of first treatment and should prompt re-evaluation of how one conducts clinical and epidemiological research in this field.

### Results in context of existing literature

We found only two prior systematic reviews describing obesity prevalence in patients receiving systemic anticancer therapy, and both involved clinical trial populations with slight variations in the cancer types under evaluation.^15^^,^^16^ Findings were consistent, though, on which cancer types had lower and higher levels of obesity at first systemic therapy—notably lower for gastrointestinal cancers and higher in breast cancer. Pestine et al.^15^ reported a median obesity prevalence of 10% across pancreatic cancer studies and a median of 30% across breast cancer studies, aligning with our respective estimates of 13% and 34% (Table 2). Vaidya et al.^16^ reported increasing obesity prevalence in the United States trials of breast cancer patients from 25.5% in 1986 to 47.5% in 2016. The most recent estimate of 47.5% is substantially higher than our obesity prevalence estimate of 34% in breast cancer; however, this disparity is likely explained by differences in the background population rates of obesity in the United States (most recently estimated to be >40%^36^ versus England, which is approaching 30%^32^).

Multiple lines of evidence support the plausibility of our findings; however, we recognise this study is one population-based assessment in isolation, and the results are not contextualised against individuals without cancer. Thus, our obesity prevalence estimates may be specific only to patients undergoing systemic anticancer therapy in England. A systematic review aimed at gathering and synthesising obesity proportions reported in baseline tables of prior published cohorts of patients treated with systemic anticancer therapies outside of the trial setting and across countries would provide greater certainty on the generalizability of our analysis and reveal international variation.

### Strengths and limitations

A study strength was the ability to measure obesity (defined as BMI ≥30 kg/m^2^) in our target population with fewer exclusions than typically observed in a real-world EHR-based analysis. Weight and height inform systemic therapy dosing and therefore are routinely measured in cancer patients and entered into the SACT dataset; 83% of patients had a valid BMI measurement at first treatment. We observed that missing or implausible BMI values were more frequent for some cancer types, older individuals, males, and in certain regions of England. Regional differences in recording practices have been known since the inception of the SACT dataset and can stem from the use of electronic prescribing systems or lack thereof.^23^ Further explorations revealed therapies prescribed as fixed (and not weight-based) doses as a reason for missingness, including endocrine therapy, commonly used in older patients with breast cancer and males with prostate cancer, explaining the variation in BMI completeness across age, sex and cancer types. Age standardisation would have reduced the influence of age-based differences but does not completely eliminate the possibility of some residual selection bias. While BMI completeness improved over time (Supplementary Table S2), results should also be interpreted acknowledging the completeness of systemic treatments recorded in the SACT dataset. Endocrine therapies again are known to be under-captured because they are often delivered outside of hospitals and instead in primary care in England.^23^ Overall, we expect that missingness in BMI and SACT treatment data is unlikely to bias comparisons between cancer types but may affect absolute prevalences in some subgroups.

Unlike many EHR studies, which rely upon specifying time windows to select study variable information (months or even years around a date of interest), we had BMI values corresponding exactly to our study index date (first systemic regimen), increasing measurement precision. General practitioners (GPs) in England also routinely record weight, height and BMI at patient registration and at NHS health checks, inviting individuals aged 40-74 years every 5 years^37^ or annually for those with chronic conditions. Previous GP records research suggests that within the general English population, BMI is remeasured infrequently but, perhaps conveniently for our question, more often in individuals who are overweight.^38^ As we considered all valid historic BMI values, calculating the maximal for each patient, it is likely that if an individual had a clinically relevant history of obesity, it would have been recorded in QResearch over time and captured in our lifetime obesity prevalence definition. We cannot, however, exclude that some patients with a history of obesity might have been misclassification or missed, in particular during the COVID-19 pandemic when anthropometric measures were less often captured in GP records. We also did not distinguish duration, timing or severity of obesity exposure, nor metabolically healthy versus metabolically unhealthy obesity; therefore, the interpretation of lifetime obesity prevalence is limited to whether an individual has ever been exposed to obesity.

BMI is often criticised as an imprecise measure of excess adiposity, and redefining what constitutes obesity has been the topic of a recent Lancet commission involving 58 multidisciplinary experts. Differentiating preclinical from clinical obesity, the commission outlined 18 diagnostic criteria for the clinical manifestation and effect of obesity on body physiology,^39^ which may well be irreversible. Our study offers further insight into the issue of single time point BMI, which fails to reflect past obesity exposure and its prognostic implications. Longitudinal BMI helped us overcome cross-sectional data limitations, and although the Lancet commission discredit the use of BMI alone to distinguish obesity in individuals, they acknowledge the importance of BMI in population-level epidemiological studies, further defending its use in this study.

As it was not prespecified, our main analysis did not use the recently recommended lower obesity threshold of ≥27.5 kg/m^2^ in black, Asian and other minority ethnicity groups. Our sensitivity analysis of ethnicity-adjusted BMI cut-offs found this made little difference to the prevalence of obesity observed at first treatment in the overall sample; however, there was a 15 percentage point increase in obesity prevalence in patients from black, Asian and other minority ethnicity groups, which is nontrivial and matches with other research impact assessments.^33^

### Future research directions: impact of GLP-1 receptor agonists

Our study underlines the importance of using longitudinal BMI measures to accurately classify obesity exposure in cancer patients receiving systemic therapy, and subsequent research should examine how lifetime maximal BMI versus BMI at treatment start associates with cancer outcomes to answer the obesity paradox with the view to improve cancer prognostication. A future challenge in understanding this complex relationship will be the increasing availability and exposure of patients to GLP-1 and dual GIP/GLP-1 receptor agonists and other treatments in development for use in diabetes and weight loss,^40^ and arguably, longitudinal BMI measures will become even more important.

In our sample of patients receiving systemic anticancer therapy who were diagnosed and treated in England during 2013 and 2023, there were <1% of patients with record of receiving a GLP-1 receptor agonist. Time will show if expanded GLP-1 use reduces obesity in patients receiving systemic anticancer therapy; we will understand the benefits and risks of these drugs used in conjunction with systemic therapy and determine whether GLP-1 receptor agonists positively impact upon cancer outcomes. Our study therefore provides much needed obesity prevalence data in a population very minimally exposed to weight loss interventions for examining change over time.

### Conclusion

We found that approximately one in four cancer patients in England were living with obesity at the start of systemic treatment, while half had a history of obesity. Reliance on BMI at treatment initiation for cancer prognostication substantially underestimates lifetime exposure to obesity, with implications for precision medicine and outcomes research.
