# Supplementary material for: Real-world obesity prevalence and history in 79 271 patients receiving systemic anticancer therapy across 13 cancer types in England (2013-2023)
Source: ESMO Real World Data Digit Oncol. 2026 Apr 24;12:100700. doi: 10.1016/j.esmorw.2026.100700 (PMC13129467; doi:10.1016/j.esmorw.2026.100700)
Supplement: Supplementary Material [file mmc1.pdf]

Supplemental Methods

Figure S1. Venn Diagram of systemic anticancer therapy patients identified in one or more data source: cancer registry, hospital episode statistics, or QResearch (2013-2020)

Table S1. Body mass index data management rules

Supplemental Results

Table S2. Age distributions of systemic anticancer therapy patients included and excluded from the analysis, by cancer type

Table S3. Study sample characteristics at systemic therapy start, by cancer type

Figure S2. Observed obesity prevalence by cancer type and age group, sex, ethnicity, deprivation and region of England

Figure S3. Observed obesity prevalence by cancer type and sex

Figure S4. Observed obesity prevalence by cancer type and ethnicity

Figure S5. Observed obesity prevalence by cancer type and deprivation quintile

Figure S6. Observed obesity prevalence by cancer type region of England

Figure S7. Observed prevalence at first treatment and history of obesity for the overall sample of systemic anticancer therapy patients, by subgroups of age, sex, ethnicity, deprivation and region of England

Table S4. Observed obesity prevalence at treatment start for the overall sample of systemic anticancer therapy patient without/with ethnicity adjusted body mass index thresholds for obesity

Table S5. Observed obesity prevalence at treatment start for the overall sample of systemic anticancer therapy patient without/with exclusion of patients with second cancers within 5 years

Supplemental Methods

Figure S1. Venn Diagram of systemic anticancer therapy patients identified in one or more data source: cancer registry, hospital episode statistics, or QResearch (2013-2020)

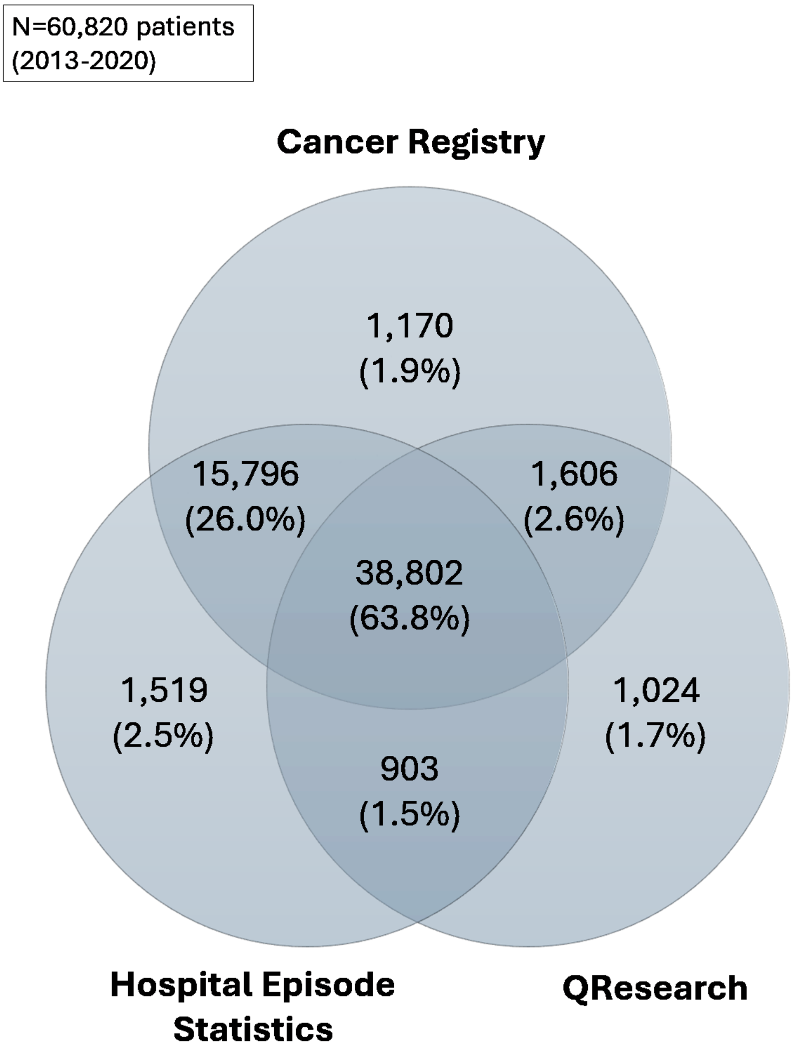

As the national cancer registrations dataset was censored at the end of 2020, diagnoses of cancer beyond this date were identified from QResearch and HES records only. As shown in the above Venn diagram, based on data from the years 2013-2020 (when all three sources of cancer diagnosis information were available), only n=1,170 (1.9%) patients had their cancer diagnosis recorded in the national cancer registrations dataset only and in no other data source. Hence, assuming the same would be true for the years 2021-2023, we expect that approximately 98% of all eligible systemic anticancer patients would have been included in the analysis.

Table S1. Body mass index data management rules

**BMI Definition**	**SACT dataset**<br>→BMI was calculated from recorded values of weight in kilograms and height in metres (kg/m^2)<br>→Where >1 BMI value was available on the same date, 1 value was selected for analysis at random
	**QResearch**<br>→BMI was either directly recorded in QResearch or calculated from recorded values of weight in kilograms and height in metres (kg/m^2)<br>→Calculated BMI values used weight and height values recorded on the same date in the first instance, and otherwise used weight values, together with the height value recorded on the nearest available date<br>→Where >1 BMI value was available on the same date, 1 value was selected for analysis at random
**Exclusions**	<u>BMI values were excluded if **incomplete:**</u><br>Recorded BMI value missing or one or both components of recorded weight and height missing<br><u>BMI values were excluded if **implausible**:</u><br>Weight < 20 kg<br>Height <1.21m or >2.14m<br>BMI <5 kg/m^2 or >200 kg/m^2<br><u>BMI values were excluded if **inconsistent**:</u><br>>1 recorded BMI value available on the same day that differed by more than 0·5 kg/m^2<br>Difference between recorded and calculated BMI on the same day that differed by more than 1.0 kg/m^2<br><u>BMI values were excluded if recorded:</u><br><u>**in pregnancy**</u><br><u>**in childhood (<18 years)**</u><br><u>**after start of first systemic therapy**</u>

Longitudinal body mass index (BMI) measures were either directly recorded in QResearch or calculated from recorded values of weight in kilograms and height in metres (kg/m^2) in QResearch and SACT. BMI values were managed using rules developed in prior peer-reviewed research using electronic health records and involved excluding incomplete, implausible and inconsistent values, as well as values recorded during childhood and pregnancy.[1,2]

References

1. Bhaskaran K, et al. Representativeness and optimal use of body mass index (BMI) in the UK Clinical Practice Research Datalink (CPRD). BMJ Open. 2013 Sep 13;3(9):e003389.
2. Katsoulis M, et al. Identifying adults at high-risk for change in weight and BMI in England: a longitudinal, large-scale, population-based cohort study using electronic health records. Lancet Diabetes Endocrinol. 2021 Oct;9(10):681-694.

Supplemental Results

Table S2. Completeness of valid BMI values in patients with valid first systemic therapy record in the SACT dataset across patient characteristics (1/2)

		N	Complete BMI, n (% row)
**Patients with valid SACT record**	All patients	95,456	79,271 (83.0%)
**Age group at treatment start**	18 to 54 years	19,517	16,704 (85.6%)
	55 to 64 years	22,506	19,420 (86.3%)
	65 to 74 years	31,110	25,823 (83.0%)
	75+ years	22,323	17,324 (77.6%)
**Sex**	Female	50,205	42,936 (85.5%)
	Male	45,251	36,335 (80.3%)
**Ethnicity**	White	85,346	70,584 (82.7%)
	Indian	1,310	1,127 (86.0%)
	Pakistani	827	698 (84.4%)
	Bangladeshi	472	423 (89.6%)
	Other Asian	939	820 (87.3%)
	Caribbean	1,220	1,034 (84.8%)
	Black African	1,242	1,077 (86.7%)
	Chinese	347	303 (87.3%)
	Other	2,338	2,036 (87.1%)
	Missing	1,415	1,169 (82.6%)
**Townsend deprivation fifth**	1 (least deprived)	30,498	25,197 (82.6%)
	2	23,473	19,470 (82.9%)
	3	17,253	14,345 (83.1%)
	4	13,405	11,015 (82.2%)
	5 (most deprived)	10,354	8,846 (85.4%)
	Missing	473	398 (84.1%)
**Region**	East Midlands	2,035	1,526 (75.0%)
	East of England	3,967	3,318 (83.6%)
	London	17,196	15,356 (89.3%)
	North East	3,080	2,235 (72.6%)
	North West	22,976	17,935 (78.1%)
	South Central	11,894	11,102 (93.3%)
	South East	10,565	8,993 (85.1%)
	South West	9,885	7,707 (78.0%)
	West Midlands	11,046	8,951 (81.0%)
	Yorkshire & Humber	2,812	2,148 (76.4%)
**Cancer type**	Breast	22,687	18,859 (83.1%)
	Bowel	16,266	14,831 (91.2%)
	Lung	15,276	13,298 (87.1%)
	Non-Hodgkin Lymphoma	8,377	7,425 (88.6%)
	Gastroesophageal	7,062	6,349 (89.9%)
	Prostate	9,263	5,441 (58.7%)
	Pancreas	3,458	3,185 (92.1%)
	Ovarian	3,602	3,140 (87.2%)
	Bladder	3,489	2,532 (72.6%)
	Malignant melanoma	2,403	1,723 (71.7%)
	Kidney	2,274	1,505 (66.2%)
	Hepatocellular	781	511 (65.4%)
	Uterine	518	472 (91.1%)

Table S2. Completeness of valid BMI values in patients with valid first systemic therapy record in the SACT dataset across patient characteristics (2/2)

		N	Complete BMI, n (% row)
**Year of first treatment**	2013	5,133	3,001 (58.5%)
	2014	7,209	4,957 (68.8%)
	2015	7,662	5,726 (74.7%)
	2016	8,612	6,992 (81.2%)
	2017	9,488	7,824 (82.5%)
	2018	10,488	9,256 (88.3%)
	2019	11,043	9,830 (89.0%)
	2020	10,548	9,251 (87.7%)
	2021	11,287	9,981 (88.4%)
	2022	11,260	10,073 (89.5%)
	2023	2,726	2,380 (87.3%)
**First SACT record treatment type**	Chemotherapy, immunotherapy, and targeted therapy	87,880	75,997 (86.5%)
	Endocrine therapy, and supportive therapies	7,576	3,274 (43.2%)

Table S3. Study sample characteristics at systemic therapy start, by cancer type (1/2)

Patient characteristic at systemic therapy start		Breast N=18,859	Prostate N=5,441	Lung N=13,298	Bowel N=14,831	Melanoma N=1,723	Kidney N=1,505	Pancreas N=3,185	Bladder N=2,532
**Age at treatment start**	median (25th, 75th)	56.7 (48.4, 66.5)	72.2 (66.4, 77.4)	69.1 (62.1, 74.9)	66.7 (58.3, 74.0)	68.3 (57.8, 76.3)	67.4 (58.9, 74.2)	68.5 (60.6, 74.7)	71.4 (64.1, 77.0)
**Age group, n (%)**	18 to 54 years	8553 (45.4)	127 (2.3)	1274 (9.6)	2609 (17.6)	342 (19.8)	232 (15.4)	385 (12.1)	209 (8.3)
	55 to 64 years	4939 (26.2)	977 (18.0)	3235 (24.3)	4001 (27.0)	366 (21.2)	412 (27.4)	808 (25.4)	479 (18.9)
	65 to 74 years	3710 (19.7)	2372 (43.6)	5507 (41.4)	4996 (33.7)	508 (29.5)	530 (35.2)	1227 (38.5)	978 (38.6)
	75+ years	1657 (8.8)	1965 (36.1)	3282 (24.7)	3225 (21.7)	507 (29.4)	331 (22.0)	765 (24.0)	866 (34.2)
**Sex, n (%)**	female	18740 (99.4)	--	5978 (45.0)	6226 (42.0)	722 (41.9)	476 (31.6)	1457 (45.7)	630 (24.9)
	male	119 (0.6)	5441 (100)	7320 (55.0)	8605 (58.0)	1001 (58.1)	1029 (68.4)	1728 (54.3)	1902 (75.1)
**Ethnicity, n (%)**	White	16088 (85.3)	4884 (89.8)	12199 (91.7)	13291 (89.6)	1658 (96.2)	1379 (91.6)	2845 (89.3)	2369 (93.6)
	Indian	386 (2.0)	61 (1.1)	102 (0.8)	174 (1.2)	<10 NR	22 (1.5)	45 (1.4)	21 (0.8)
	Pakistani	273 (1.4)	23 (0.4)	69 (0.5)	94 (0.6)	<10 NR	14 (0.9)	17 (0.5)	<20 NR
	Bangladeshi	120 (0.6)	<10 NR	73 (0.5)	81 (0.5)	<10 NR	<10 NR	16 (0.5)	<20 NR
	Other Asian	287 (1.5)	24 (0.4)	104 (0.8)	119 (0.8)	<10 NR	13 (0.9)	41 (1.3)	24 (0.9)
	Caribbean	299 (1.6)	115 (2.1)	145 (1.1)	169 (1.1)	<10 NR	14 (0.9)	55 (1.7)	<20 NR
	Black African	397 (2.1)	110 (2.0)	100 (0.8)	202 (1.4)	<10 NR	12 (0.8)	43 (1.4)	<20 NR
	Chinese	92 (0.5)	<10 NR	50 (0.4)	63 (0.4)	<10 NR	<10 NR	14 (0.4)	<10 NR
	Other	711 (3.8)	121 (2.2)	266 (2.0)	355 (2.4)	23 (1.3)	23 (1.5)	62 (1.9)	36 (1.4)
	Missing	206 (1.1)	90 (1.7)	190 (1.4)	283 (1.9)	25 (1.5)	21 (1.4)	47 (1.5)	30 (1.2)
**Deprivation fifth, n (%)**	Q1 (least deprived)	5886 (31.2)	2067 (38.0)	3282 (24.7)	5066 (34.2)	695 (40.3)	500 (33.2)	1059 (33.2)	843 (33.3)
	Q2	4679 (24.8)	1385 (25.5)	2968 (22.3)	3724 (25.1)	471 (27.3)	380 (25.2)	770 (24.2)	634 (25.0)
	Q3	3448 (18.3)	868 (16.0)	2589 (19.5)	2578 (17.4)	265 (15.4)	302 (20.1)	582 (18.3)	466 (18.4)
	Q4	2570 (13.6)	602 (11.1)	2308 (17.4)	1949 (13.1)	175 (10.2)	173 (11.5)	403 (12.7)	346 (13.7)
	Q5 (most deprived)	2151 (11.4)	486 (8.9)	2098 (15.8)	1447 (9.8)	101 (5.9)	147 (9.8)	358 (11.2)	237 (9.4)
	Missing	125 (0.7)	33 (0.6)	53 (0.4)	67 (0.5)	16 (0.9)	<10 NR	13 (0.4)	<10 NR
**Region, n (%)**	East Midlands	350 (1.9)	138 (2.5)	254 (1.9)	291 (2.0)	29 (1.7)	29 (1.9)	54 (1.7)	53 (2.1)
	East of England	767 (4.1)	251 (4.6)	430 (3.2)	629 (4.2)	60 (3.5)	58 (3.9)	143 (4.5)	150 (5.9)
	London	3975 (21.1)	956 (17.6)	2646 (19.9)	2775 (18.7)	272 (15.8)	235 (15.6)	668 (21.0)	401 (15.8)
	North East	526 (2.8)	95 (1.7)	490 (3.7)	349 (2.4)	24 (1.4)	63 (4.2)	78 (2.4)	148 (5.8)
	North West	4322 (22.9)	1189 (21.9)	3492 (26.3)	3283 (22.1)	385 (22.3)	317 (21.1)	704 (22.1)	547 (21.6)
	South Central	2558 (13.6)	939 (17.3)	1618 (12.2)	2085 (14.1)	317 (18.4)	244 (16.2)	452 (14.2)	376 (14.8)
	South East	2141 (11.4)	565 (10.4)	1346 (10.1)	1734 (11.7)	217 (12.6)	167 (11.1)	386 (12.1)	277 (10.9)
	South West	1738 (9.2)	529 (9.7)	1262 (9.5)	1397 (9.4)	195 (11.3)	156 (10.4)	283 (8.9)	249 (9.8)
	West Midlands	2009 (10.7)	642 (11.8)	1332 (10.0)	1891 (12.8)	179 (10.4)	185 (12.3)	335 (10.5)	264 (10.4)
	Yorkshire & Humber	473 (2.5)	137 (2.5)	428 (3.2)	397 (2.7)	45 (2.6)	51 (3.4)	82 (2.6)	67 (2.6)

Table S3. Study sample characteristics at systemic therapy start, by cancer type (2/2)

Patient characteristic at systemic therapy start		Gastroesophageal N=6,349	Ovarian N=3,140	Hepatocellular N=511	Uterine N=472	Non-Hodgkin Lymphoma N=7,425
**Age at treatment start**	median (25th, 75th)	68.1 (60.2, 74.4)	66.7 (56.7, 74.9)	67.8 (60.6, 73.9)	67.1 (58.4, 73.2)	69.0 (58.6, 76.8)
**Age group, n (%)**	18 to 54 years	821 (12.9)	656 (20.9)	66 (12.9)	85 (18.0)	1345 (18.1)
	55 to 64 years	1646 (25.9)	762 (24.3)	140 (27.4)	118 (25.0)	1537 (20.7)
	65 to 74 years	2443 (38.5)	954 (30.4)	195 (38.2)	174 (36.9)	2229 (30.0)
	75+ years	1439 (22.7)	768 (24.5)	110 (21.5)	95 (20.1)	2314 (31.2)
**Sex, n (%)**	female	1717 (27.0)	3140 (100)	121 (23.7)	472 (100)	3257 (43.9)
	male	4632 (73.0)	--	390 (76.3)	--	4168 (56.1)
**Ethnicity, n (%)**	White	5759 (90.7)	2789 (88.8)	418 (81.8)	376 (79.7)	6529 (87.9)
	Indian	64 (1.0)	76 (2.4)	13 (2.5)	14 (3.0)	142 (1.9)
	Pakistani	40 (0.6)	30 (1.0)	16 (3.1)	<10 NR	100 (1.3)
	Bangladeshi	40 (0.6)	15 (0.5)	<10 NR	<10 NR	54 (0.7)
	Other Asian	55 (0.9)	39 (1.2)	16 (3.1)	12 (2.5)	83 (1.1)
	Caribbean	86 (1.4)	32 (1.0)	<10 NR	21 (4.4)	72 (1.0)
	Black African	64 (1.0)	29 (0.9)	10 (2.0)	14 (3.0)	78 (1.1)
	Chinese	20 (0.3)	20 (0.6)	<10 NR	<10 NR	24 (0.3)
	Other	127 (2.0)	67 (2.1)	16 (3.1)	16 (3.4)	213 (2.9)
	Missing	94 (1.5)	43 (1.4)	<10 NR	<10 NR	130 (1.8)
**Deprivation fifth, n (%)**	1 (least deprived)	1937 (30.5)	1085 (34.6)	128 (25.0)	149 (31.6)	2500 (33.7)
	2	1563 (24.6)	832 (26.5)	105 (20.5)	104 (22.0)	1855 (25.0)
	3	1206 (19.0)	513 (16.3)	99 (19.4)	90 (19.1)	1339 (18.0)
	4	917 (14.4)	434 (13.8)	89 (17.4)	65 (13.8)	984 (13.3)
	5 (most deprived)	701 (11.0)	258 (8.2)	87 (17.0)	64 (13.6)	711 (9.6)
	Missing	25 (0.4)	18 (0.6)	<10 NR	<10 NR	36 (0.5)
**Region, n (%)**	East Midlands	105 (1.7)	56 (1.8)	<10 NR	<10 NR	151 (2.0)
	East of England	234 (3.7)	152 (4.8)	23 (4.5)	14 (3.0)	407 (5.5)
	London	1158 (18.2)	578 (18.4)	165 (32.3)	132 (28.0)	1395 (18.8)
	North East	185 (2.9)	68 (2.2)	<10 NR	10 (2.1)	197 (2.7)
	North West	1493 (23.5)	640 (20.4)	94 (18.4)	73 (15.5)	1396 (18.8)
	South Central	842 (13.3)	455 (14.5)	58 (11.4)	56 (11.9)	1102 (14.8)
	South East	755 (11.9)	371 (11.8)	74 (14.5)	53 (11.2)	907 (12.2)
	South West	600 (9.5)	344 (11.0)	34 (6.7)	60 (12.7)	860 (11.6)
	West Midlands	821 (12.9)	378 (12.0)	45 (8.8)	57 (12.1)	813 (10.9)
	Yorkshire & Humber	156 (2.5)	98 (3.1)	<10 NR	<10 NR	197 (2.7)

Notes: Deprivation is based on the Townsend score, divided in fifths from 1 to 5, where 1 corresponds to the 'least' and 5 corresponds the 'most' deprived areas of England. For statistical disclosure control, table cells with fewer than 10 patients are not reported (censored). **Abbreviations:** NR = not reported

Figure S2. Observed obesity prevalence by cancer type and age group

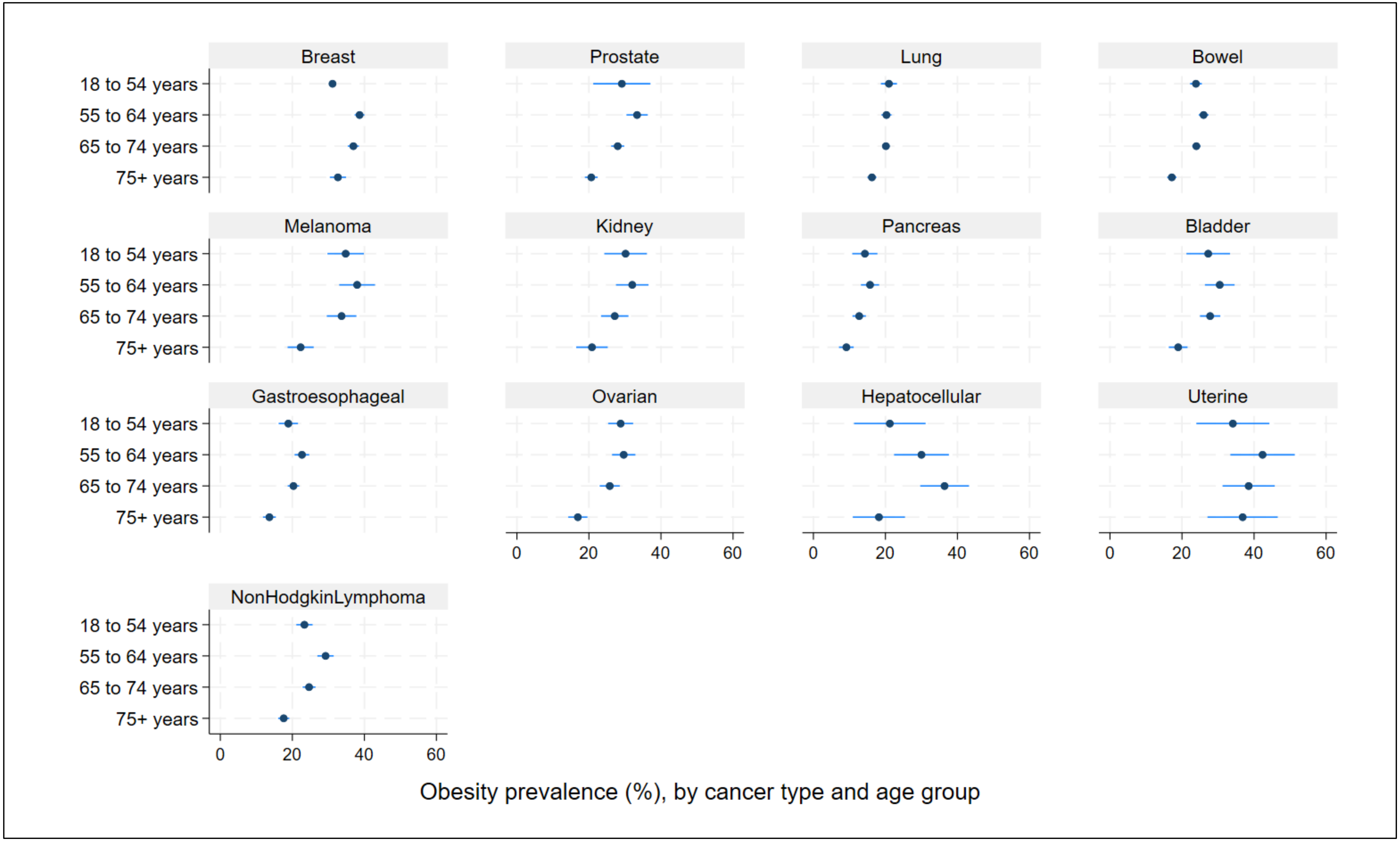

Notes: Plot shows obesity prevalence (dark blue dot) with 95% confidence interval (light blue error bar).

Figure S3. Observed obesity prevalence by cancer type and sex

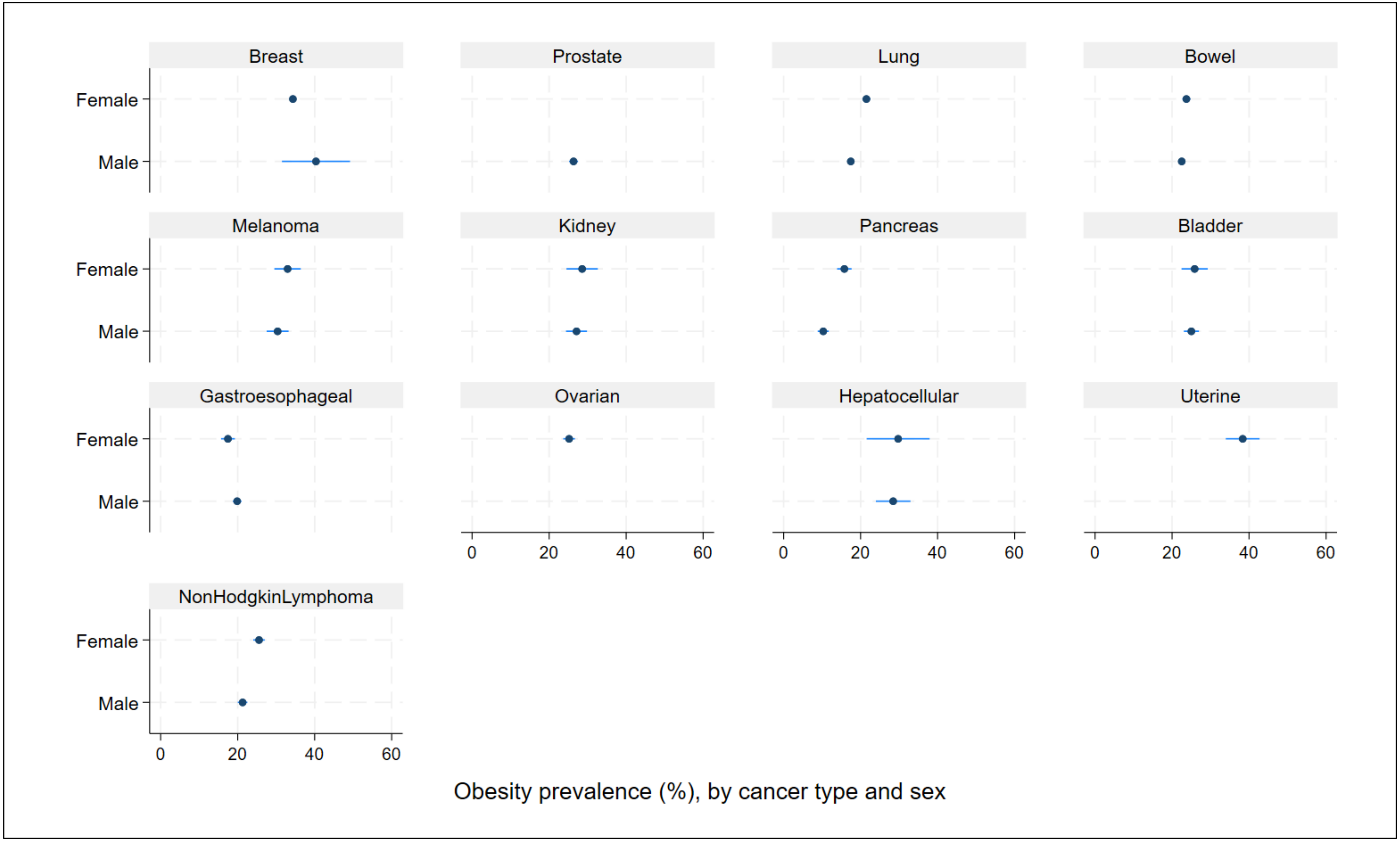

Notes: Plot shows obesity prevalence (dark blue dot) with 95% confidence interval (light blue error bar).

Figure S4. Observed obesity prevalence by cancer type and ethnicity

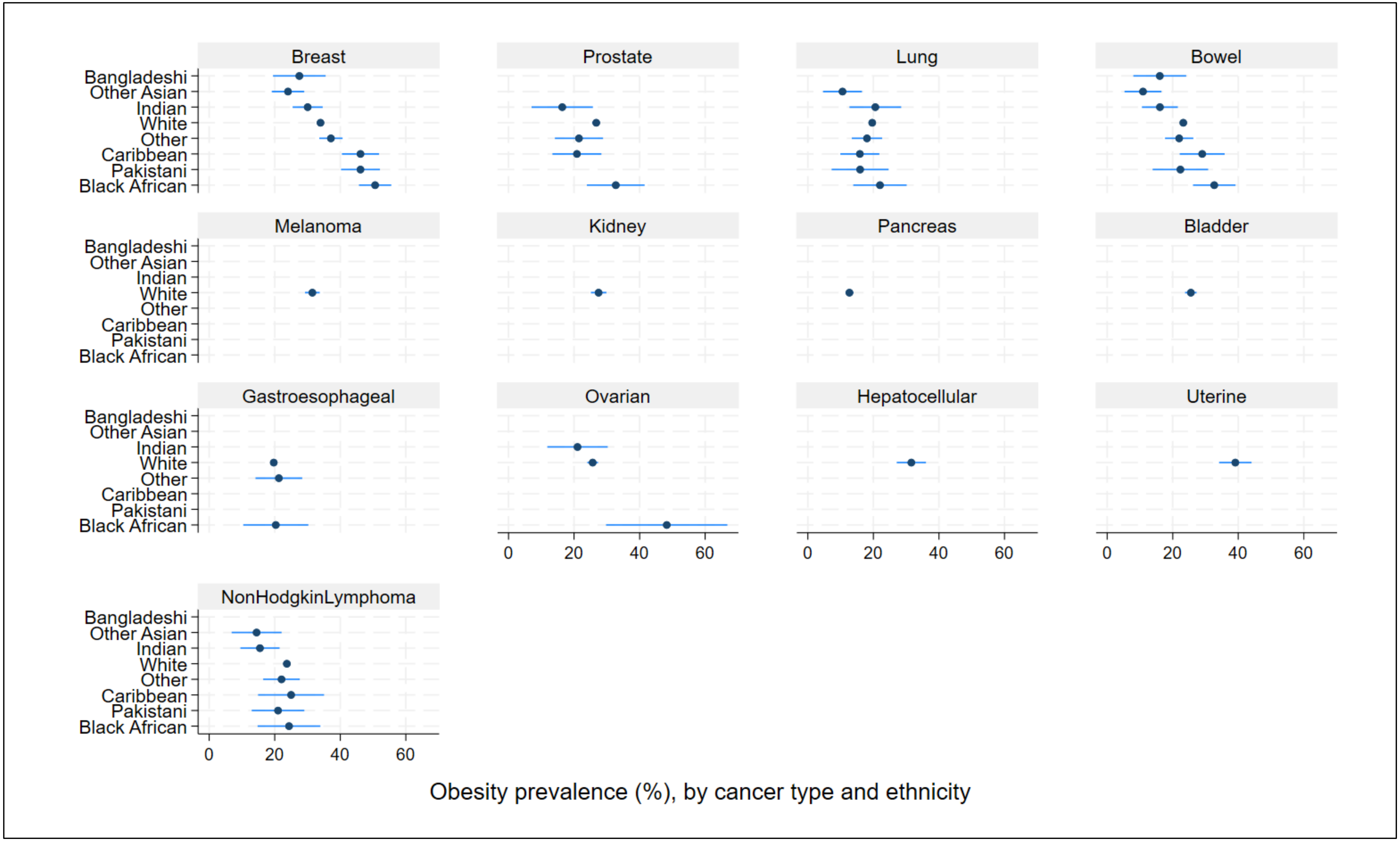

Notes: Plot shows obesity prevalence (dark blue dot) with 95% confidence interval (light blue error bar). Frequency counts with fewer than 10 patients with obesity, and corresponding percentages, are not reported.

Figure S5. Observed obesity prevalence by cancer type and deprivation fifth

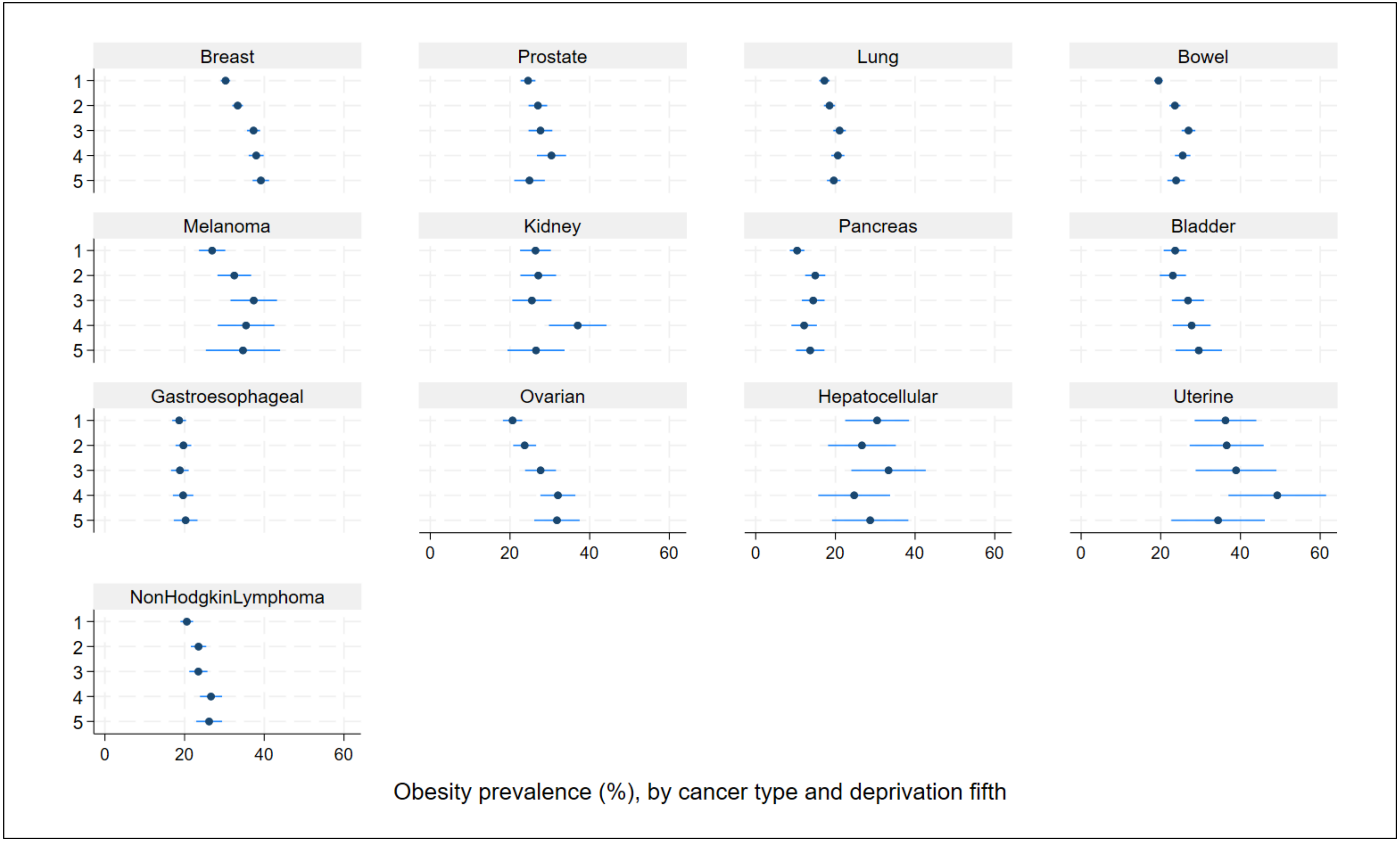

Notes: Plot shows obesity prevalence (dark blue dot) with 95% confidence interval (light blue error bar). Deprivation is based on the Townsend score, divided in from fifths 1 to 5, where 1 corresponds to the 'least' and 5 corresponds the 'most' deprived areas of England.

Figure S6. Observed obesity prevalence by cancer type and region of England

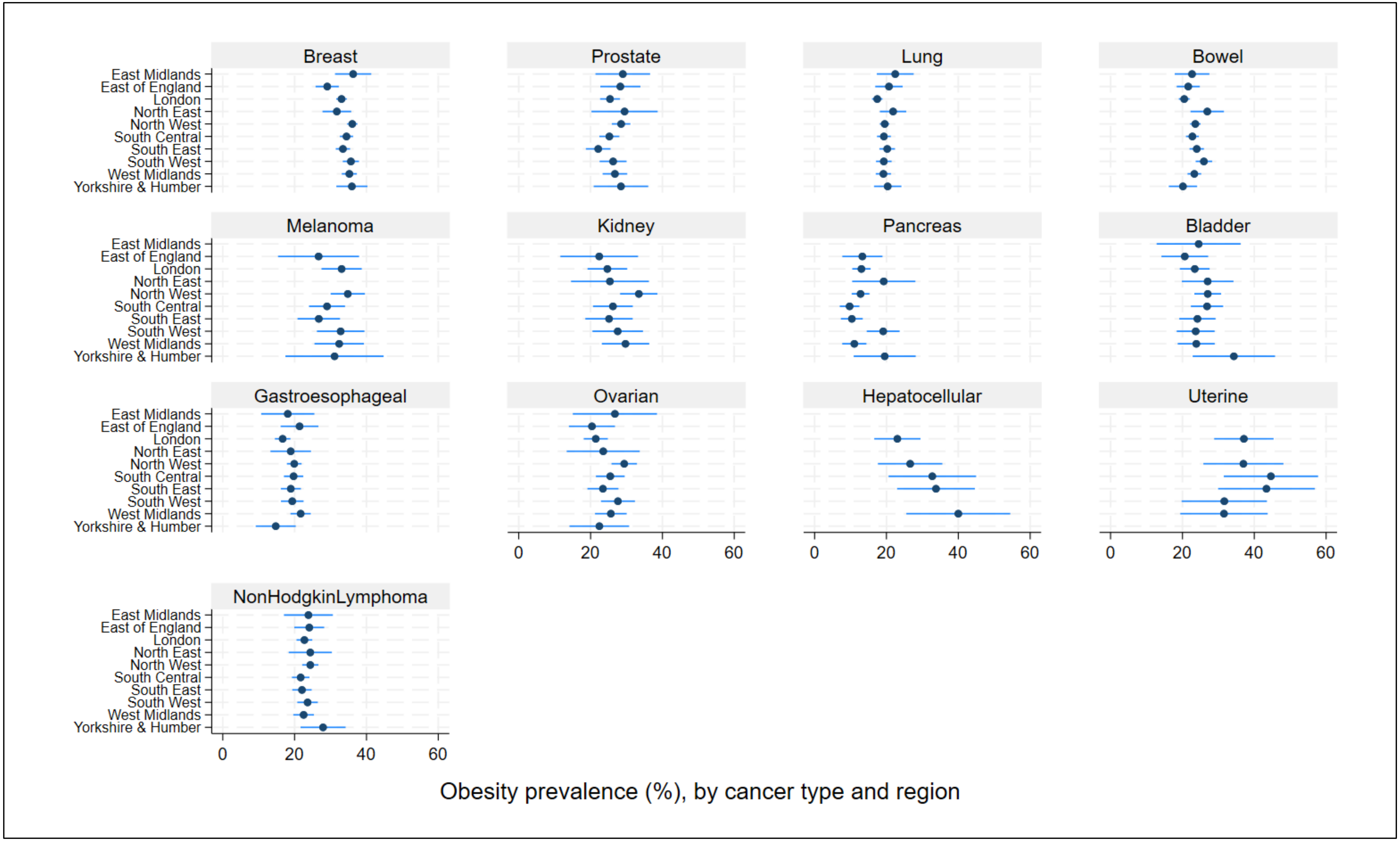

Notes: Plot shows obesity prevalence (dark blue dot) with 95% confidence interval (light blue error bar). Frequency counts with fewer than 10 patients with obesity, and corresponding percentages, are not reported.

Figure S7. Observed prevalence at first treatment and history of obesity for the overall sample of systemic anticancer therapy patients, by subgroups of age, sex, ethnicity, deprivation and region of England

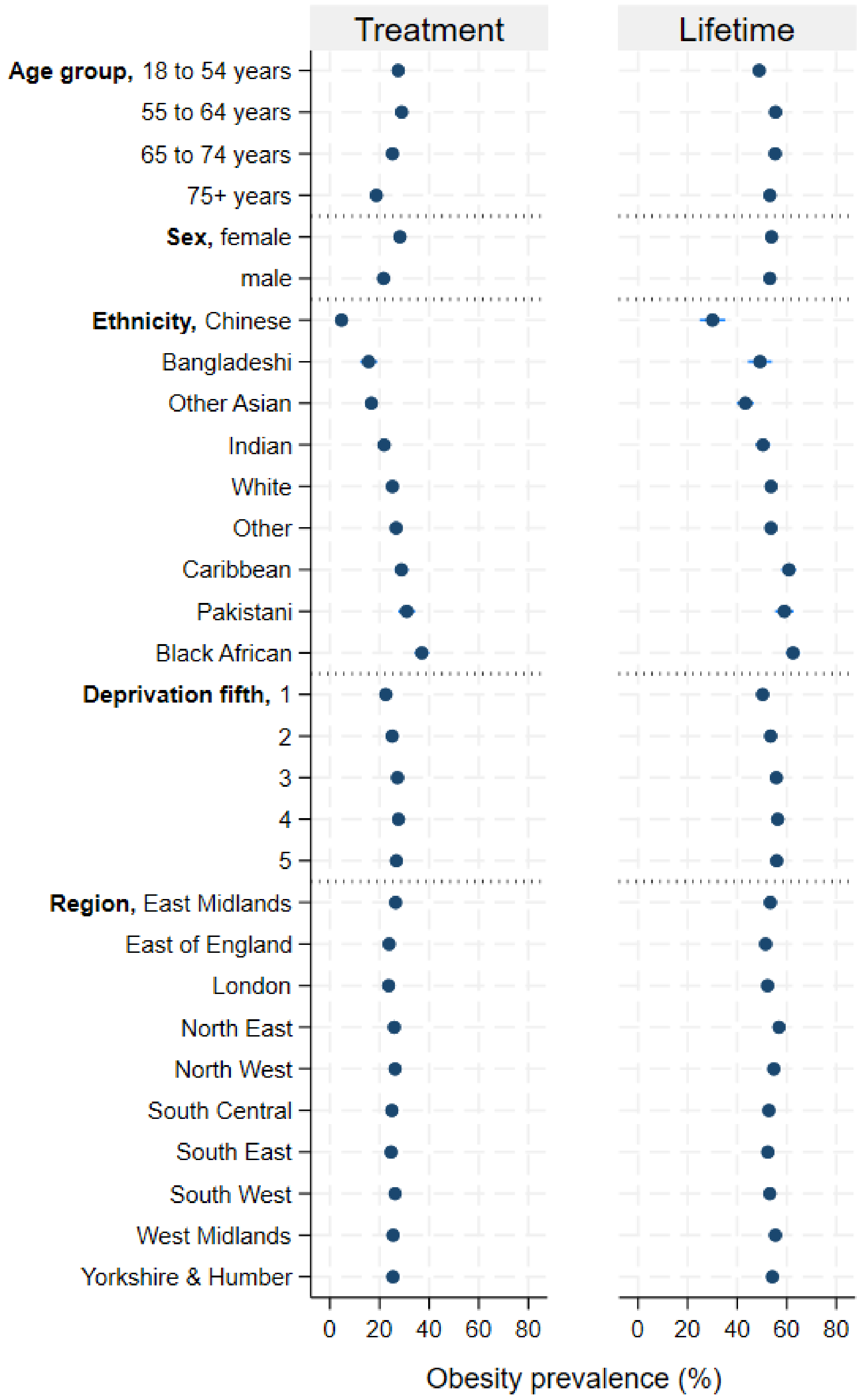

Notes: Plot shows obesity prevalence (dark blue dot) with 95% confidence interval (light blue error bar). Deprivation is based on the Townsend score, divided in fifths from 1 to 5, where 1 corresponds to the 'least' and 5 corresponds the 'most' deprived areas of England.

Table S4. Observed obesity prevalence at treatment start for the overall sample of systemic anticancer therapy patient without/with ethnicity adjusted body mass index thresholds for obesity

	Without ethnicity adjustment		With ethnicity adjustment	
	n / N	Obesity prevalence	n / N	Obesity prevalence
All patients	19,958/79271	25.2% [24.9-25.5%]	20,799/79,271	26.2% [25.9-26.5%]
Black, Asian and Other minority ethnicity group	1,376/5,482	25.1% [24.0-26.2%]	2217/5482	40.4% [39.1-41.7%]
Indian	246/1127	21.8% [19.4-24.2%]	418/1127	37.1% [34.3-39.9%]
Pakistani	216/698	30.9% [27.5-34.4%]	341/698	48.9% [45.1-52.6%]
Bangladeshi	66/423	15.6% [12.1-19.1%]	136/423	32.2% [27.7-36.6%]
Other Asian	137/820	16.7% [14.2-19.3%]	240/820	29.3% [26.2-32.4%]
Caribbean	298/1034	28.8% [26.1-31.6%]	468/1034	45.3% [42.2-48.3%]
Black African	399/1077	37.0% [34.2-39.9%]	575/1077	53.4% [50.4-56.4%]
Chinese	14/303	4.6% [2.3-7.0%]	39/303	12.9% [9.1-16.6%]

Table S5. Observed obesity prevalence at treatment start for the overall sample of systemic anticancer therapy patient without/with exclusion of patients with second cancers within 5 years

	Second cancers included		Second cancers excluded	
	n / N	Obesity prevalence	n / N	Obesity prevalence
All patients	19,958/79,271	25.2% [24.9-25.5%]	18,531/73,114	25.3% [25.0-25.7%]
Uterine	181/472	38.3% [34.0-42.7%]	157/375	41.9% [36.9-46.9%]
Melanoma	5,42/1,723	31.5% [29.3-33.6%]	483/1,440	33.5% [31.1-36.0%]
Breast	6,484/18,859	34.4% [33.7-35.1%]	6,231/18,130	34.4% [33.7-35.1%]
Kidney	415/1,505	27.6% [25.3-29.8%]	327/1,201	27.2% [24.7-29.7%]
Prostate	1,433/5,441	26.3% [25.2-27.5%]	1,266/4,585	27.6% [26.3-28.9%]
Ovarian	791/3,140	25.2% [23.7-26.7%]	657/2,599	25.3% [23.6-27.0%]
Bladder	639/2,532	25.2% [23.5-26.9%]	520/1,972	26.4% [24.4-28.3%]
Hepatocellular	147/511	28.8% [24.8-32.7%]	135/469	28.8% [24.7-32.9%]
Non-Hodgkin Lymphoma	1,719/7,425	23.2% [22.2-24.1%]	1,614/6,954	23.2% [22.2-24.2%]
Bowel	3,415/14,831	23.0% [22.3-23.7%]	3,166/13,706	23.1% [22.4-23.8%]
Lung	2,564/13,298	19.3% [18.6-20.0%]	2,440/12,692	19.2% [18.5-19.9%]
Gastroesophageal	1,220/6,349	19.2% [18.2-20.2%]	1,142/5,948	19.2% [18.2-20.2%]
Pancreas	408/3,185	12.8% [11.6-14.0%]	393/3,043	12.9% [11.7-14.1%]
